# Assessment of Cardiac Autonomic Function by Short-Term Sensor-Based and Long-Term Heart Rate Variability Analyses in Individuals with Spinal Cord Injury After Long-Term Table Tennis Training

**DOI:** 10.3390/s24227167

**Published:** 2024-11-08

**Authors:** Georgia Vogiatzi, Vasiliki Michou, Nikos Malliaropoulos, Vasileios Tsimaras, Asterios Deligiannis, Evangelia Kouidi

**Affiliations:** 1 Sports Medicine Laboratory, School of Physical Education and Sport Science, Aristotle University, 57001 Thessaloniki, Greece; vogiatzigogo@yahoo.gr (G.V.); vasilikimichou@yahoo.gr (V.M.); adeligia@phed.auth.gr (A.D.); 2 Centre Sports and Exercise Medicine, Queen Mary University of London, London E1 4DG, UK; n.malliaropoulos@qmul.ac.uk; 3 Laboratory of Motor Behaviour and Adapted Physical Activity, School of Physical Education and Sports Sciences, Aristotle University of Thessaloniki, 57001 Thessaloniki, Greece; tsimaras@phed.auth.gr

**Keywords:** spinal cord injury, tetraplegia, exercise, table tennis, autonomic nervous system, functional capacity

## Abstract

This study aimed to examine the acute and chronic effects of an exercising table tennis program on cardiac Autonomic Nervous System (ANS) and functional capacity in people with tetraplegia. Twenty males with tetraplegia (C6–C7), with a mean age of 38.50 ± 4.04 years old, were randomly assigned into two equal groups: A, who followed a 6-month exercise training program with table tennis 3 times per week, and B, who remained untrained. Additionally, 11 healthy sedentary men (group C) with a mean age of 39.71 ± 5.87 years old participated in the study as healthy controls. At baseline, all participants underwent a short-term (5 min) and a long-term (24 h ambulatory) ECG monitoring to evaluate the heart rate variability (HRV) indices and a maximal arm ergometric and dynamometric testing of the upper limbs. Moreover, the acute cardiac autonomic responses to maximal arm cycle exercise test were evaluated by Polar S810i sensor chest strap. At the end of the 6-month study, all parameters were revaluated only in groups A and B. At baseline, there was no statistically significant difference between the two patient groups. However, intra-group changes at the end of the 6-month study regarding the 24-h HRV monitoring indicated that group A statistically increased the standard deviation of R-R intervals (SDNN) by 13.9% (*p* = 0.007), the standard deviation of R-R intervals calculated every 5 min (SDANN) by 8.4% (*p* = 0.007), the very low frequency (VLF) by 7.1% (*p* = 0.042), and the low frequency [LF (ms^2^)] by 10.5% (*p* = 0.009), which almost reached the levels of group C. Favorable improvements were also noticed at the end of the study for group A in maximal exercise time of the upper limbs by 80.4% (*p* < 0.001) and maximal strength of the right hand by 27.8% (*p* < 0.001). Linear regression analysis after training showed that maximal exercise time was positively correlated with SDNN (r = 0.663, *p* = 0.036) and with LF (ms^2^) (r = 0.623, *p* = 0.045). Our results indicate that a 6-month table tennis training program is efficient and can improve cardiac ANS activity mainly by increasing sympathovagal balance.

## 1. Introduction

Cardiovascular diseases are a common cause of death for people with spinal cord injury (SCI), with a prevalence ranging from 25% to 50%, which is higher compared to non-disabled adults [1]. Dysfunction of the cardiac autonomic nervous system (ANS) is a common complication of SCI. This condition typically involves decreased activity of the cardiac sympathetic system and increased activity of the vagal system. SCI disrupts the autonomic circuits, leading to a breakdown in the coordinated functioning of both ANS branches and inadequate regulation of cardiovascular functions [2,3]. The impact on cardiac autonomic control is greater when anatomical injury occurs higher in the spinal cord, specifically in the cervical or upper thoracic region. Cervical or high-thoracic SCI is commonly associated with lifelong systemic arterial pressure control abnormalities. Resting arterial pressure is consistently lower than that of individuals with mid-to-low thoracic injuries or uninjured controls, highlighting the long-term effects of SCI [4,5].

Due to this ANS dysfunction, individuals with SCI present cardiovascular, pulmonary, gastrointestinal, musculoskeletal, neurological, and dermatological complications. Among the musculoskeletal disorders caused by SCI, pain is the most prevalent symptom of upper extremity dysfunction [6]. Inadequate muscle strength, muscle endurance, and reduced range of motion of the shoulder joint are suggested to be its causes [7]. Another significant effect of SCI is the loss of metabolically active tissue and reduced capacity to engage in aerobic exercise routinely. The level and extent of SCI affect cardiorespiratory fitness, which decreases by approximately 5% with each level of injury from T11 to C4 [8]. As a result, individuals with high-level injuries have lower cardiorespiratory fitness by about 40% compared to their non-disabled peers [8,9]. Furthermore, individuals with SCI demonstrate adaptability in coping with the overestimated resting metabolic rate, which is 5% to 32% higher than the general population. This represents 65% of the total energy expenditure [10]. The lack of active muscle groups below the level of injury likely causes a decrease in resting metabolic rate. Reduced physical activity and mobility due to SCI contribute to an increased risk of metabolic syndrome, which is linked to a higher risk of coronary artery disease [11].

Exercise plays a vital therapeutic role for individuals with SCI. It highlights the critical work of healthcare professionals and researchers in understanding and addressing these individuals’ needs. Their contributions are essential in discovering appealing and alternative forms of exercise that can encourage greater participation of people with SCI in exercise programs [6,12,13,14,15,16]. The American College of Sports Medicine has formulated comprehensive guidelines for exercise programs in individuals with SCI based on the level of injury. Engaging in 3–5 weekly exercise sessions, each lasting 20 to 60 min, with an intensity set at 50% to 80% of the individual’s maximum heart rate is recommended. These sessions can include various activities such as aerobic exercise, wheelchair propulsion, swimming, more intense exercises, wheelchair sports, walking with a partner or with crutches, seated aerobic exercise programs, and the use of an electronic limb ergometer [7]. Even though the effects of long-term exercise training programs have been thoroughly investigated in individuals with paraplegia, there is a lack of evidence regarding the impact of systemic exercise training on cardiac autonomic dysfunction in subjects with tetraplegia. Thus, this study aimed to examine the acute and chronic effects of an exercise table tennis program on cardiac ANS and functional capacity in people with SCI (level of injury C6–C7). Our study’s novelty is based on the exercise type as wheelchair table tennis, a Paralympic sport since the inaugural games in 1960, has not been the subject of any studies investigating the impact of table tennis exercise on cardiac ANS in patients with cervical SCI.

## 2. Materials and Methods

### 2.1. Participants

At first, participants with SCI were recruited from SCI associations and public sports medicine websites and were screened for eligibility. Participants’ inclusion criteria were as follows: the presence of tetraplegia (C6–C7), age > 18 years old, and male gender. Participants aged < 18 years old, receiving medication that affects the regulation of heart rate (such as b-blockers, etc.), and having previous participation in a structured exercise training program were excluded. The study also included 11 healthy age-matched individuals in the healthy control group.

### 2.2. Study Design

Initially, participants who met the inclusion criteria underwent a clinical examination [including a review of their personal and family medical history, anthropometric (weight and height) measurements, blood pressure assessment, and a recording of a 12-lead electrocardiogram (ECG) to exclude changes suggestive of ischemia and arrhythmias], a short-term (5 min) and a 24 h ambulatory ECG monitoring for heart rate variability (HRV) analysis, a dynamometry to determine the muscle strength of upper limbs, and a maximal hand-ergometric exercise testing. After baseline measurements, which took place in November 2021, participants with SCI were randomly assigned (on 17 November 2021) to either an exercise (A group) or a control group (B group). The www.randomizer.org (accessed on 7 October 2024) website was used for the randomization process. Group A followed a 6-month exercise training program with table tennis three times per week, while group B avoided participation in any structured exercise program during the study period. In addition, 11 healthy individuals formed a second control group, group C. All baseline assessments were repeated in the follow-up period only for groups A and B. The same researchers conducted all the above measurements, blinded to group allocation. This randomized controlled trial protocol was approved by the Ethics Committee of the Aristotle University of Thessaloniki (Protocol number 85/2021), ensuring the study’s ethical conduct. All participants received all necessary study information and provided written informed consent before enrolling. The clinical trial started in December 2021 and ended in June 2022.

### 2.3. 24-h Holter Monitoring

To evaluate the cardiac ANS activity, a three-channel 24 h Holter recording of ECG (SEER 1000, GE Healthcare, Chalfont St Giles, UK) was used. Subjects were advised to avoid vigorous exercise on the day of the recording, as well as the consumption of alcoholic beverages and caffeine. The ECG recording data were stored on the recorder’s memory card, and the analysis was analyzed using the Cardioday software (GE Healthcare, Chalfont St Giles, UK).

Concerning HRV time-domain analysis, four variables were assessed [17]:

- The standard deviation of R-R (the time intervals between two successive heartbeats) intervals (SDNN).
- The standard deviation of R-R intervals calculated every 5 min (SDANN).
- The square root of the mean sum of the squares of the differences between consecutive intervals R-R (rMSSD).
- The percentage of successive RR intervals higher than 50 ms (pNN50).

Similarly, concerning frequency domain analysis, five variables were assessed [17]:

- The total frequency power (TP).
- The very low-frequency power (VLF) (<0.003–0.04 Hz).
- The low-frequency power (LF) (0.04–0.15 Hz).
- The high-frequency power (HF) (0.15–0.4 Hz).
- The frequency ratio (LF/HF)

Regarding the reflection of the HRV time and frequency domains in cardiac sympathetic and parasympathetic activity, the indices are divided as follows:

- VLF and LF reflect the heart’s sympathetic activity,
- pNN50, rMSSD, and HF represent the heart’s parasympathetic activity,
- SDNN, SDANN, and the LF/HF ratio have been used to index sympathovagal activity [17].

### 2.4. POLAR S810i HRV Resting and Exercise Monitoring

The cardiac ANS activity during arm cycle ergometric exercise was also assessed with the portable heart rate monitor POLAR S810i (Polar Electro Oy, Kempele, Finland) sensor chest strap device, which collected ECGs at rest and during exercise and captured series of RR intervals for analysis of HRV indexes. The indices calculated were the rMSSD, the pNN50, the HF, the LF, and the LF/HF ratio.

### 2.5. Dynamometric Testing

The muscle strength of the upper limbs was measured using the hydraulic hand dynamometer (Baseline, New York, NY, USA). The handle of the dynamometer was individually adjusted to the size of the upper limbs of each subject so that the angle of the wrist with the instrument ranged from 0 to 30 degrees. For the correct and reliable performance of the tests, subjects had to have their upper limb at a right angle without touching their trunk [18,19]. Each measurement was preceded by a trial attempt to familiarize the participants with the instrument. Three 3 s maximal efforts were performed on the right and left upper limbs for each test. There was a 30 s break between each attempt, with the average effort measured in pounds. Likewise, the strength of the index and thumb fingers was tested.

### 2.6. Arm Cycle Ergometric Exercise Testing

Participants were carefully briefed on the examination procedure and encouraged to empty their bladders and bowels beforehand for their comfort. They then underwent a maximal arm cycle ergometric exercise test with a progressively increasing load to assess upper extremity endurance, total exercise time, and maximal heart rate. The tests were conducted using a hand ergometer (type Monark, 818E, Varberg, Sweden). The examination of maximum fatigue was conducted under the supervision of a cardiologist. The rotation speed of this manual ergometer was adjusted to the test’s requirements, and it could handle loads of up to 100 watts/min, ensuring the safety of the participants throughout the test. Blood pressure was measured before, during, and after the test. Each test started with a 2 min warm-up without resistance, followed by a fatigue protocol that gradually increased the load until exhaustion, all while maintaining a consistent pedal rotation frequency of 50 revolutions per minute with the help of a metronome (50 rpm) throughout the measurement [20,21]. The test was completed when objective and subjective indicators of maximal fatigue occurred or when some of the internationally valid criteria for stopping the test dropped in systolic blood pressure despite increasing the load, angina pectoris, symptoms from the central nervous system (dizziness, pre-fainting state), signs of reduced peripheral circulation, technical problems, or the desire of subject to interruption.

### 2.7. Table Tennis Training Program

Group A followed a 6-month table tennis training program for 6 months, three times per week. This exercise program occurred in a public gym under the supervision and instructions of the same qualified trainer and according to the International Paralympic Committee (IPC) regulations for table tennis wheelchair users: https://www.paralympic.org/ipc (assessed on 17 November 2021). Each exercise session was about 90 min long and was divided into three parts: a 10 min warm-up-stretching part, a 70 min part with medium intensity (50–70% of maximum heart rate), table tennis training, learning the basic table tennis movements (forehand, backhand, serve, chop, smash), and a 10 min recovery part with breathing and stretching exercises.

### 2.8. Statistical Analysis

For the analysis and extraction of results, the IBM SPSS 27 statistical package was used (IBM Corp. Released 2020. IBM SPSS Statistics for Windows, Version 27.0. Armonk, NY, USA). The variables were tested for normal distribution using the Kolmogorov–Smirnov test. Results are expressed as means ± standard deviation for normally distributed variables. Two-way ANOVA tested mean differences over time and between the three groups with repeated measures. A *t*-test for independent samples was also used to examine the differences between the groups. Linear regression was used to study the association between variables. The level of statistical significance was set at *p* ≤ 0.05.

## 3. Results

### 3.1. Participants Characteristics

**Figure 1:**
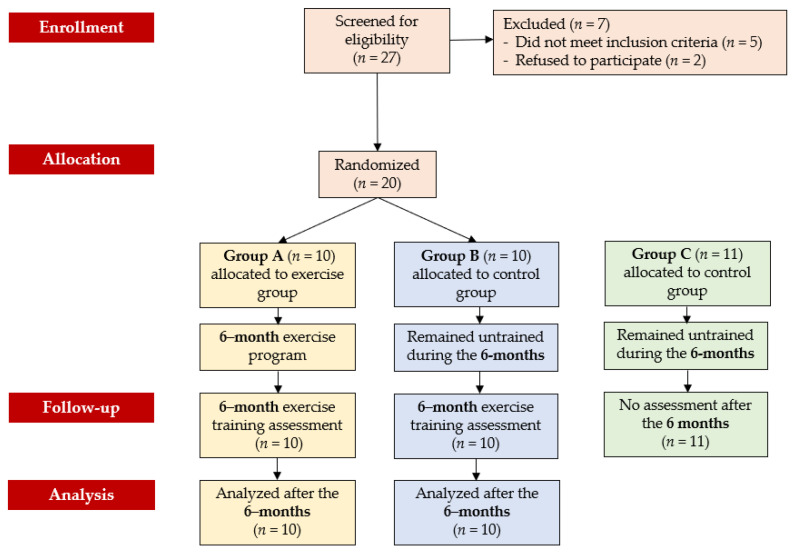
CONSORT diagram of the study design.

Initially, 27 participants with tetraplegia (C6–C7) were screened for eligibility. Five did not meet the inclusion criteria, and two declined to participate. No exercise-induced complications related to exercise and no withdrawals for personal or health reasons were observed during the study and the follow-up period. Therefore, 20 individuals with SCI and 11 healthy subjects completed the study (Figure 1). Demographic and clinical data of all participants are shown in Table 1.

### 3.2. 24 h Holter Monitoring

At baseline, there were no statistical differences in HRV time and frequency-domain indices between groups A and B. However, group C showed statistically significantly higher values in ΤP by 52.7% (*p* < 0.001), SDNN by 26.1% (*p* < 0.001), SDANN by 23.2% (*p* < 0.001), VLF by 54.2% (*p* < 0.001), LF (ms^2^) by 47.3% (*p* < 0.001), and HF (ms^2^) by 67.4% (*p* < 0.001) compared to group A. Group A results from HRV analysis after the 6-month table tennis exercise program showed significant improvements in SDNN by 13.9% (*p* = 0.007), SDANN by 8.4% (*p* = 0.007), VLF by 7.1% (*p* = 0.042), LF (ms^2^) by 10.5% (*p* = 0.009), and LF (n.u.) by 6.8% (*p* = 0.002), which almost reached the levels of group C. Regarding the inter-group changes between the A and B groups at the end of the study, results for the A group showed a favorable increase in SDNN by 15.1% (*p* = 0.001), SDANN by 7.7%% (*p* = 0.033), VLF by 7.1% (*p* = 0.001), LF (ms^2^) by 10.1% (*p* = 0.046), and LF (n.u.) by 7.3% (*p* = 0.023) (Table 2).

### 3.3. POLAR S810i HRV Resting and Exercise Monitoring

After the 6-month table tennis exercise program, group A showed a significant increase in resting LF (n.u.) by 8.2% (*p* < 0.001), resting LF/HF ratio by 12.2% (*p* = 0.002), exercise LF (n.u.) by 8.4% (*p* = 0.001), and exercise LF/HF ratio by 49.8% (*p* < 0.001). In contrast, group A reduced resting HF (n.u.) by 7.8% (*p* = 0.008), exercise rMSSD by 11.8% (*p* = 0.002), exercise pNN50 by 26.6% (*p* = 0.037), and exercise HF (n.u.) by 31.2% (*p* < 0.001). Regarding the inter-group changes between the A and B groups at the end of the study, results for the A group showed a favorable increase in resting LF/HF ratio by 11.5% (*p* = 0.002), exercise LF (n.u.) by 6.2% (*p* = 0.029), and exercise LF/HF ratio by 51.0% (*p* = 0.026), while lower values were noticed in exercise pNN50 (decrease by 24.7%, *p* = 0.035) and exercise HF (n.u.) (decrease by 30.4%, *p* = 0.001). In addition, group C showed statistically significant values at resting and exercising HRV, compared to group A, both at baseline and after 6 months (Table 3).

### 3.4. Dynamometric Testing Results

All participants had the right hand as the dominant one. At baseline, there were no statistically significant differences between groups A and B. In contrast, group A showed reduced initial values in terms of the right-hand strength by 61.7% (*p* < 0.001), the left hand by 68.4% (*p* < 0.001), the right finger by 39.3% (*p* < 0.001), and the left finger by 39.6% (*p* < 0.001) compared to group C. After the 6-month study, group A showed a significant increase only in strength of the right hand by 27.8% (*p* < 0.001). Regarding the inter-group changes between the A and B groups at the end of the study, results for the A group showed a favorable increase in the strength of the right hand by 34.6% (*p* = 0.019). While inter-group changes between A and C groups were demonstrated, group A showed a reduced strength in the right hand by 51.1% (*p* < 0.001) and in the left hand by 64.9% (*p* < 0.001), as well as in the right finger by 37.7% (*p* < 0.001) and left finger by 35.7% (*p* < 0.001) (Table 4).

### 3.5. Arm Cycle Ergometric Fatigue Testing Results

At baseline, there were no statistical differences in hand-ergometric fatigue testing indices between groups A and B. However, group C showed statistically significant higher values in resting HR by 23.7% (*p* = 0.002), exercise time by 64.9% (*p* < 0.001), maximum load by 53.5% (*p* < 0.001), and maximum HR by 30.4% (*p* < 0.001) compared to group A. After the 6-month study, group A showed favorable improvements in exercise time (increase of 80.4%, *p* < 0.001) and maximum load (increase of 69.2%, *p* < 0.001). Regarding the inter-group changes between the A and B groups at the end of the study, results for the A group showed a significant increase in exercise time by 80.4% (*p* < 0.001) and maximum load by 59.4% (*p* < 0.001). In addition, although the fatigue test demonstrated a higher resting HR (increase by 23.0%, *p* = 0.003), longer exercise time (increase by 36.7%, *p* = 0.022) and a higher maximum HR (increase by 27.1%, *p* < 0.001) in group C at the end of the study, group A did not show a statistically significant difference in maximum load and Borg’s scale score compared to group C (Table 5).

### 3.6. Linear Regression Analysis

**Figure 2:**
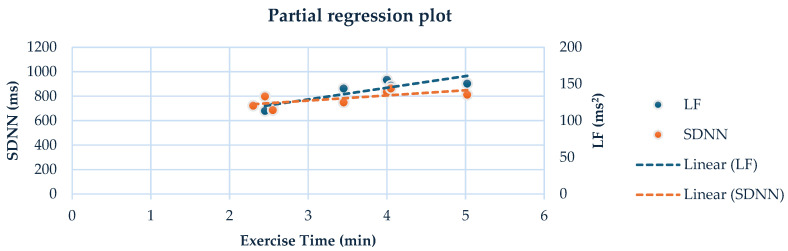
Linear regression analysis between the exercise time and LF (ms^2^) (r = 0.623, *p* = 0.045), and between exercise time and SDNN (ms) (r = 0.663, *p* = 0.036) in the group A, by using the 24-h HRV analysis.

At the end of the study, a positive correlation was noticed in group A between exercise time (min) and LF (ms^2^) (r = 0.623, *p* = 0.045), and between exercise time and SDNN (r = 0.663, *p* = 0.036), by using the 24-h HRV analysis (Figure 2).

## 4. Discussion

Our study revealed that a 6-month table tennis training program yielded positive cardiac ANS and functional capacity outcomes in individuals with cervical SCI. Significant improvements in time and frequency domain indices after acute and long-term exercise via Polar S810i sensor chest strap device and 24-h HRV monitoring were observed, respectively. Additionally, the program improved upper extremity strength, exercise time, and maximum load. To our knowledge, this study is the first to investigate long-term table tennis training programs’ effects on the cardiac ANS activity of individuals with tetraplegia. Delving into the impact of exercise on individuals with SCI, particularly the intricate action of the ANS on the heart, is a complex and intriguing journey. The altered function of the heart’s sympathetic and parasympathetic nervous systems and the resulting imbalance caused by the injury add a layer of complexity that keeps us engaged in this fascinating topic.

In general, the cranial parasympathetic nervous system remains intact after an SCI at any neurological level. However, sacral parasympathetic function is permanently compromised. In contrast, the sympathetic nervous system can be affected to varying degrees, depending on both the injury’s neurological level and the completeness of the autonomic damage [22]. These modifications directly result from SCI and may account for increased susceptibility to cardiovascular disease [23]. Damage to the ANS’s spinal and/or central components results in impaired cardiac and blood vessel neural control [24]. Cardiac sympathetic nerve fibers innervating the heart originate from the thoracic cord between T1 and T5. Consequently, individuals with SCI above the T6 spinal segment experience impaired or absent cardiovascular sympathetic control [25].Thus, most individuals with SCI above T6 experience daily persistent hypotension and bradycardia, as well as episodic drops in blood pressure when assuming an upright posture [25,26]. Furthermore, transient episodes of aberrantly low and high blood pressure can be life-threatening, leading to clinical complications such as orthostatic hypotension and autonomic dysreflexia [27]. In addition, HRV, a non-invasive tool for assessing cardiac autonomic control, is markedly impacted after SCI. Research has also indicated a decrease in HRV is observed within 24 months after SCI. This fact suggests that the decline is partly attributable to the direct impact of SCI, rather than solely a long-term consequence of living with SCI [28].

Systematic exercise is highly important in regulating the cardiac ANS in SCI patients. Unfortunately, only a few studies have examined the effects of exercise on cardiac ANS in individuals with SCI. These studies included mainly individuals with paraplegia and subjects with low cardiovascular risk factors [7,26]. In patients with cervical SCI, the maximal HR response to exercise ranges between 100 and 120 bpm, while those with thoracic SCI show a relatively average maximal HR [29,30,31]. The reduced HR response to maximal exercise, observed in subjects with cervical SCI, is due to decreased cardiac sympathetic activity and lower levels of circulating catecholamines. However, they still experience a slight increase in noradrenaline’s levels during exercise, possibly due to spillover from continuously active postganglionic sympathetic nerve endings [32,33,34]. Notably, the exercise-induces increase in noradrenaline’s levels is significantly lower in individuals with cervical SCI than those with low-thoracic SCI and healthy individuals [33,34]. In our study, at baseline, we observed that the healthy controls significantly increased maximum HR by 30.4% compared to group A with cervical SCI in the arm cycle ergometric fatigue test. This difference existed at the end of the study for our exercised patients, emphasizing the differences in the hemodynamic responses to exercise among healthy individuals and patients with cervical SCI. Thus, the decreased maximum HR in cervical SCI reflects a dependence on vagal inhibition of the sinoatrial node and a slight exercise-induced rise in circulating noradrenaline [22].

In addition, our study showed that long-term table tennis training improved the HRV response to acute arm cycle ergometric exercise, measured by a Polar S810i sensor monitor, in group A. Our trained individuals demonstrated positive improvements in time and frequency domain indices at rest and during exercise. Studies in healthy people have shown that this sensor is a cost-effective and practical device. It has been used to monitor beat-to-beat heart rate for HRV analysis, providing a consistent RR series that can be used for HRV analysis during both rest and exercise [35,36]. Moreover, Gamelin et al. [37] showed that the Polar S810 has a 0.4% rate error at rest compared to the ECG, while Vanderlei et al. [38] found no statistically significant difference between the Polar S810i device and the ECG at rest or during exercise. In general, the Polar S810 sensor has good to near-perfect validity as a measure of short-term HRV [36].

Similarly to our results, Romero et al. [39], by using the Polar RS800 HR (Polar Electro, Kempele, Finland) observed significant differences between the SCI group (lesion level between C5–C7) and healthy controls after the end of an acute arm cycle ergometer session for the variables SDNN, rMSSD, HF, and LF at rest, 5 and 10 min after the exercise with the highest values found in individuals without SCI. While, Wecht et al. [40], in an attempt to determine HRV acute response to aerobic exercise via an arm ergometer in individuals with SCI (lesion level below T6), showed a significant decrease in LF, HF and LF/HF ratio immediately after exercise. These findings suggest that the ANS’s response in individuals with tetraplegia during exercise is similar to that of healthy individuals but with lower index values [35]. However, acute HRV response has also been investigated in other exercise modalities. Interestingly, Takahashi et al. [31], found that patients with tetraplegia (injury level between C6–C7) experienced a decrease in LF and HF immediately after static arm exercise. These values returned to normal levels within 90 s. In another study, a reduction in LF was observed 5 min after handgrip exercise, with no changes in HF [41].

Furthermore, our study revealed that long-term table tennis training improved 24-h HRV indices reflecting sympathovagal activity, leading to a consequent sympathovagal balance after six months. However, inter-group changes at the end of the study indicated that group A statistically increased SDNN, SDANN, LF (ms^2^), LF (n.u.), and decreased HF (ms^2^), compared to group B. Meanwhile, group C had higher values in both time and frequency domain variables than group A. According to the literature, few studies have focused on the impact of exercise programs on the ANS in individuals with quadriplegia. These exercise programs with the use of an arm ergometer had a mean duration of 8 to 10 weeks, with 20–30-min sessions three times per week and an intensity equal to 50% to 70% of the maximum HR [42,43]. Notably, there was no control group in any study. In these studies, upper extremity exercise for individuals with SCI aimed also to enhance upper extremity muscle strength and endurance. Thus, results of these studies have also shown favorable improvements in exercise time and muscle strength [21,42,43].

Similarly, our study revealed significant improvements within group A in exercise time, maximum load, maximum HR, and strength of the right hand following an arm cycle ergometric and a dynamometric test at the end of the study. Based on the results, we conclude that table tennis exercise significantly improved upper extremity strength endurance, apparently due to the movements and techniques of the sport performed by people with quadriplegia. These findings have practical implications, suggesting that table tennis exercise can significantly improve upper extremity strength endurance in individuals with SCI, thereby enhancing their daily lives and quality of life.

Additionally, at the end of the 6-month study, a medium positive correlation was found between exercise time and SDNN and between exercise time and LF (ms^2^). In general, table tennis significantly strains the cardiovascular system [44]. Healthy athletes exert substantial effort during significant events such as the Olympics, World Championships, and Pan-European games. To date, only one study compared HRV indices before and after playing a table tennis simulated competitive match in healthy subjects [45]. However, the heart rate has been examined in other tasks in this sport, such as in serve kinematics and multiball training [46,47]. In contrast, no study has examined the effects of table tennis on HRV in SCI patients, highlighting the scientific value of our study in this population.

To sum up, it’s important to acknowledge this study’s strengths and limitations. Firstly, this study has revealed significant exercise-induced improvements in HRV, muscle strength, and functional capacity. Secondly, it is the first study to assess the effects of a long-term table tennis training program on the cardiac ANS in individuals with tetraplegia and revealed significant correlations between exercise time and time- and frequency- domain indices. However, the study has notable limitations, including the exclusion of female participants. This was primarily due to the potential influence of menstrual cycle symptoms, like lower abdominal pain, on the study protocol. As a result, females were less likely to participate consistently throughout the 6-month duration than males. Additionally, the small number of participants posed a challenge, mainly stemming from difficulties in recruiting individuals with cervical spinal cord injuries who were willing to commit to a long-term study. This is a common challenge in SCI research, and we acknowledge the need for more diverse participant populations in future studies.

## 5. Conclusions

In conclusion, the study’s findings strongly support the implementation of a carefully organized and systematic 6-month table tennis exercise program for individuals with cervical SCI. The program has been shown to be not only feasible but also safe and effective in improving the cardiac autonomic function. The adaptations to training mainly included sympathovagal balance and improvement of upper extremity fatigue time and maximum load achieved.

## Figures and Tables

**Table 1:** Demographic and clinical characteristics of participants.

	Participants with SCI		Healthy Inividuals	A vs. B	A vs. C
	Group A (*n_A_* = 10)	Group B (*n_B_* = 10)	Group C (*n_C_* = 11)	*p*-Value	*p*-Value
Age (years)	37.71 ± 4.38	38.30 ± 4.02	39.71 ± 5.87	*p* = 0.184	*p* = 0.150
Height (cm)	1.75 ± 0.05	1.76 ± 0.05	1.77 ± 0.05	*p* = 0.213	*p* = 0.402
Weight (Kg)	80.20 ± 7.88	80.70 ± 5.81	84.14 ± 6.54	*p* = 0.573	*p* = 0.134
BMI (Kg/m^2^)	26.12 ± 3.32	25.97 ± 2.50	26.45 ± 3.28	*p* = 0.347	*p* = 0.568
SCI duration (years)	12.80 ± 2.09	12.90 ± 3.28	-	*p* = 0.423	-

Note: BMI: Body Mass Index; SCI: Spinal Cord Injury; Data are expressed as mean ± SD; *p* < 0.05: group A vs. B; *p* < 0.05: group A vs. C.

**Table 2:** HRV results from 24 h heart rate recording.

	Group A			Group B			Group C	Group A vs. B		Group A vs. C	
	Baseline	After 6-Months	*p*-Value	Baseline	After 6-Months	*p*-Value	Baseline	Pre	Post	Pre	Post
HR (bpm)	57.06 ± 8.27	59.6 ± 6.20	*p* = 0.118	56.11 ± 9.33	56.03 ± 9.15	*p* = 0.352	75.11 ± 13.70	*p* = 0.817	*p* = 0.261	*p* = 0.075	*p* = 0.001 ***
TP (ms^2^)	1939.33 ± 367.51	2048.89 ± 451.70	*p* = 0.394	2000.95 ± 111.17	2000.61 ± 162.08	*p* = 0.990	4100.56 ± 714.09	*p* = 0.768	*p* = 0.754	*p* < 0.001 ***	*p* < 0.001 ***
SDNN (ms)	110.79 ± 8.89	126.26 ± 11.76	*p* = 0.007 *	109.55 ± 9.57	109.68 ± 9.38	*p* = 0.858	149.99 ± 7.16	*p* = 0.800	*p* = 0.001 **	*p* < 0.001 ***	*p* < 0.001 ***
SDANN (ms)	100.00 ± 7.04	108.35 ± 8.68	*p* = 0.007 *	100.90 ± 8.56	100.61 ± 9.05	*p* = 0.619	130.33 ± 5.29	*p* = 0.871	*p* = 0.033 **	*p* < 0.001 ***	*p* < 0.001 ***
rMSSD (ms)	42.01 ± 5.28	41.23 ± 3.14	*p* = 0.530	41.64 ± 4.75	41.31 ± 4.97	*p* = 0.457	40.80 ± 1.54	*p* = 0.979	*p* = 0.966	*p* = 0.496	*p* = 0.703
pNN50 (%)	8.24 ± 0.81	8.68 ± 1.21	*p* = 0.064	8.23 ± 0.89	8.23 ± 0.95	*p* = 1.000	11.88 ± 0.99	*p* = 0.740	*p* = 0.370	*p* < 0.001 ***	*p* < 0.001 ***
VLF (ms^2^)	816.02 ± 130.63	873.93 ± 118.72	*p* = 0.042 *	811.51 ± 118.51	816.01 ± 113.01	*p* = 0.481	1784.29 ± 402.65	*p* = 0.939	*p* = 0.001 **	*p* < 0.001 ***	*p* < 0.001 ***
LF (ms^2^)	717.31 ± 169.81	792.73 ± 136.15	*p* = 0.009 *	719.18 ± 82.23	720.43 ± 83.47	*p* = 0.742	1362.31 ± 255.73	*p* = 0.975	*p* = 0.046 **	*p* < 0.001 ***	*p* < 0.001 ***
LF (n.u.)	72.23 ± 10.29	77.16 ± 10.38	*p* = 0.002 *	71.54 ± 5.66	71.92 ± 5.57	*p* = 0.443	73.71 ± 8.36	*p* = 0.855	*p* = 0.023 **	*p* = 0.728	*p* = 0.424
HF (ms^2^)	358.02 ± 132.59	318.91 ± 133.01	*p* = 0.390	355.85 ± 42.66	354.14 ± 49.69	*p* = 0.698	1102.07 ± 137.43	*p* = 0.961	*p* = 0.443	*p* < 0.001 ***	*p* < 0.001 ***
HF (n.u.)	38.42 ± 11.87	37.71 ± 10.90	*p* = 0.698	37.52 ± 3.48	36.98 ± 5.11	*p* = 0.425	43.73 ± 3.72	*p* = 0.821	*p* = 0.850	*p* = 0.194	*p* = 0.116
LF/HF	2.12 ± 0.50	2.27 ± 0.89	*p* = 0.335	1.91 ± 0.21	2.05 ± 0.40	*p* = 0.306	1.72 ± 0.24	*p* = 0.316	*p* = 0.355	*p* = 0.164	*p* = 0.060

Note: HRV: Heart rate variability; TP: Total; RR intervals: Time intervals between two successive heartbeats; SDNN: standard deviation of RR intervals; SDANN: Standard deviation of the 5 min Average NN intervals; rMSSD: root mean square of successive differences between normal heartbeats; pNN50: The number of pairs of successive NN (R-R) intervals that differ by more than 50 ms; VLF: very low frequency; LF: Low frequency; HF: High frequency; Data are expressed as mean ± SD; * *p* < 0.05: baseline vs. 6 months follow-up; ** *p* < 0.05: group A vs. B; *** *p* < 0.05: group A vs. C.

**Table 3:** Results were derived from POLAR S810i HRV resting and exercise monitoring at baseline and the end of the study in group A.

	Group A			Group B			Group C	Group A vs. B		Group A vs. C	
	Baseline	After 6-Months	*p*-Value	Baseline	After 6-Months	*p*-Value	Baseline	Pre	Post	Pre	Post
**Resting HRV**											
rMSSD (ms)	49.94 ± 3.91	48.93 ± 3.00	*p* = 0.651	49.28 ± 5.31	49.04 ± 5.02	*p* = 0.373	40.25 ± 5.62	*p* = 0.588	*p* = 0.480	*p* = 0.817	*p* = 0.671
pNN50 (%)	31.63 ± 3.19	30.67 ± 4.54	*p* = 0.298	30.81 ± 2.29	30.55 ± 2.47	*p* = 0.653	37.14 ± 6.80	*p* = 0.893	*p* = 0.479	*p* = 0.041 ***	*p* = 0.003 ***
LF (n.u.)	52.77 ± 5.62	57.09 ± 5.41	*p* < 0.001 *	52.27 ± 3.82	51.91 ± 3.92	*p* = 0.174	126.42 ± 13.87	*p* = 0.818	*p* = 0.118	*p* < 0.001 ***	*p* < 0.001 ***
HF (n.u.)	37.64 ± 2.62	34.69 ± 2.93	*p* = 0.008 *	36.87 ± 2.51	35.61 ± 2.12	*p* = 0.061	89.01 ± 11.75	*p* = 0.794	*p* = 0.886	*p* < 0.001 ***	*p* < 0.001 ***
LF/HF	1.47 ± 0.21	1.65 ± 0.19	*p* = 0.002 *	1.42 ± 0.15	1.46 ± 0.11	*p* = 0.284	1.42 ± 0.23	*p* = 0.977	*p* = 0.002 **	*p* = 0.062	*p* = 0.002 ***
**Exercise HRV**											
rMSSD (ms)	14.57 ± 1.29	12.85 ± 1.52	*p* = 0.002 *	14.77 ± 1.03	14.68 ± 1.27	*p* = 0.494	11.56 ± 1.67	*p* = 0.964	*p* = 0.735	*p* = 0.035 ***	*p* = 0.024 ***
pNN50 (%)	0.79 ± 0.18	0.58 ± 0.17	*p* = 0.037 *	0.78 ± 0.17	0.77 ± 0.09	*p* = 0.511	0.56 ± 0.11	*p* = 0.237	*p* = 0.035 **	*p* = 0.001 ***	*p* < 0.001 ***
LF (n.u.)	60.49 ± 2.43	65.57 ± 4.05	*p* = 0.001 *	61.07 ± 4.03	61.75 ± 2.80	*p* = 0.137	187.44 ± 23.07	*p* = 0.139	*p* = 0.029 **	*p* < 0.001 ***	*p* < 0.001 ***
HF (n.u.)	26.22 ± 2.74	18.03 ± 2.52	*p* < 0.001 *	26.00 ± 2.62	25.92 ± 2.71	*p* = 0.343	36.99 ± 5.48	*p* = 0.853	*p* = 0.001 **	*p* < 0.001 ***	*p* < 0.001 ***
LF/HF	2.41 ± 0.32	3.61 ± 0.80	*p* < 0.001 *	2.36 ± 0.24	2.39 ± 0.22	*p* = 0.100	5.06 ± 0.71	*p* = 0.710	*p* = 0.026 **	*p* < 0.001 ***	*p* < 0.001 ***

Note: HRV: Heart rate variability; rMSSD: root mean square of successive differences between normal heartbeats; pNN50: The number of pairs of successive NN (R-R) intervals that differ by more than 50 ms; LF: Low frequency; HF: High frequency. Data are expressed as mean ± SD; * *p* < 0.05: baseline vs. 6 months follow-up; ** *p* < 0.05: group A vs. B; *** *p* < 0.05: group A vs. C.

**Table 4:** Dynamometric testing results.

	Group A			Group B			Group C	Group A vs. B		Group A vs. C	
	Baseline	After 6-Months	*p*-Value	Baseline	After 6-Months	*p*-Value	Baseline	Pre	Post	Pre	Post
Right hand (kg)	29.80 ± 9.01	38.10 ± 11.13	*p* < 0.001 *	28.68 ± 4.43	28.30 ± 4.47	*p* = 0.169	77.90 ± 9.64	*p* = 0.729	*p* = 0.019 **	*p* < 0.001 ***	*p* < 0.001 ***
Left hand (kg)	20.85 ± 7.95	23.15 ± 9.16	*p* = 0.084	20.10 ± 7.51	20.20 ± 7.49	*p* = 0.343	66.10 ± 11.87	*p* = 0.831	*p* = 0.441	*p* < 0.001 ***	*p* < 0.001 ***
Right fingers (kg)	15.60 ± 3.71	16.00 ± 3.16	*p* = 0.711	15.25 ± 1.96	14.80 ± 2.39	*p* = 0.330	25.70 ± 4.32	*p* = 0.795	*p* = 0.351	*p* < 0.001 ***	*p* < 0.001 ***
Left fingers (kg)	14.00 ± 1.69	14.90 ± 1.37	*p* = 0.068	13.70 ± 1.76	13.10 ± 2.23	*p* = 0.081	23.20 ± 2.52	*p* = 0.703	*p* = 0.043 **	*p* < 0.001 ***	*p* < 0.001 ***

Data are expressed as mean ± SD; * *p* < 0.05: baseline vs. 6 months follow-up; ** *p* < 0.05: group A vs. B.; *** *p* < 0.05: group A vs. C.

**Table 5:** Arm cycle ergometric fatigue testing results.

	Group A			Group B			Group C	Group A vs. B		Group A vs. C	
	Baseline	After 6-Months	*p*-Value	Baseline	After 6-Months	*p*-Value	Baseline	Pre	Post	Pre	Post
HRrest (bpm)	54.00 ± 10.86	54.50 ± 11.21	*p* = 0.863	54.40 ± 5.60	54.90 ± 4.72	*p* = 0.537	70.8 ± 10.30	*p* = 0.919	*p* = 0.918	*p* = 0.002 ***	*p* = 0.003 ***
Exercise time (min)	1.84 ± 3.32	3.32 ± 0.87	*p* < 0.001 *	1.83 ± 0.42	1.84 ± 0.39	*p* = 0.936	5.25 ± 2.27	*p* = 0.964	*p* < 0.001 **	*p* < 0.001 ***	*p* = 0.022 ***
Maximum load (watt)	6.50 ± 2.41	11.00 ± 2.10	*p* < 0.001 *	6.50 ± 2.41	6.90 ± 2.02	*p* = 0.674	14.00 ± 4.59	*p* = 1.000	*p* < 0.001 **	*p* < 0.001 ***	*p* = 0.077
HRmax (bpm)	101.80 ± 12.81	106.70 ± 7.97	*p* = 0.235	100.70 ± 11.12	101.50 ± 9.44	*p* = 0.837	146.30 ± 17.58	*p* = 0.840	*p* = 0.200	*p* < 0.001 ***	*p* < 0.001 ***
Borg’s scale score	15.30 ± 0.67	15.90 ± 1.10	*p* = 0.193	15.00 ± 0.94	14.80 ± 1.26	*p* = 0.104	15.60 ± 1.17	*p* = 0.424	*p* = 0.125	*p* = 0.492	*p* = 0.563

Note: HR: Heart rate; Data are expressed as mean ± SD; * *p* < 0.05: baseline vs. 6 months follow-up; ** *p* < 0.05: group A vs. B; *** *p* < 0.05: group A vs. C.

## Data Availability

The data presented in this study are available on request from the corresponding author. The data are not publicly available due to ethical restrictions.
